# *In vivo* imaging of axonal transport in peripheral nerves of rodent forelimbs

**DOI:** 10.1042/NS20220098

**Published:** 2023-01-19

**Authors:** Qiuhan Lang, Giampietro Schiavo, James N. Sleigh

**Affiliations:** 1 Department of Neuromuscular Diseases and UCL Queen Square Motor Neuron Disease Centre, Queen Square Institute of Neurology, University College London, London WC1N 3BG, U.K.; 2 UK Dementia Research Institute, University College London, London WC1E 6BT, U.K.

**Keywords:** axonal transport, live imaging, median nerve, motor neuron, neuromuscular junction (NMJ), ulnar nerve

## Abstract

Axonal transport is the essential process by which neurons actively traffic a variety of cargoes between the cell soma and axon terminals. Accordingly, dysfunctional axonal transport is linked to many nervous system conditions. Therefore, being able to image and quantify this dynamic process in live neurons of animal disease models is beneficial for understanding neuropathology and testing new therapies at the preclinical level. As such, intravital approaches have been developed to assess cargo movement in the hindlimb sciatic nerves of live, anaesthetised mice. Here, we describe an adapted method for *in vivo* imaging of axonal transport in intact median and ulnar nerves of the rodent forelimb. Injection of a fluorescently labelled and non-toxic fragment of tetanus neurotoxin (H_C_T) into the mouse forepaw permits the identification of signalling endosomes in intact axons of median and ulnar nerves. Through immunofluorescent analysis of forelimb lumbrical muscles and median/ulnar nerves, we confirmed that H_C_T is taken up at motor nerve terminals and predominantly locates to motor axons. We then showed that the baseline trafficking of signalling endosomes is similar between the median/ulnar nerves and the sciatic nerve in adult wild-type mice. Importantly, this adapted method can be readily tailored for assessment of additional cargoes, such as mitochondria. By measuring transport in forelimb and hindlimb nerves, comparative anatomical and functional analyses can be performed in rodent disease models to aid our understanding of peripheral nerve disease pathogenesis and response to injury.

## Introduction

Axonal transport is the process through which neurons actively and bidirectionally traffic important biological cargoes (e.g., organelles, proteins and RNAs) between the cell soma and axon terminals, and it is essential for neuronal homeostasis and survival [1]. Accordingly, dysfunctional axonal transport has been reported in many neurological diseases [2], including peripheral neuropathies [3] and amyotrophic lateral sclerosis (ALS) [4]. Furthermore, mutations in genes encoding key components of the axonal transport machinery are known to cause neurodegeneration in humans [5]. Therefore, being able to image axonal transport and quantify the dynamics of different cargoes is beneficial for understanding neuropathology and evaluating preclinical therapies [6].

Several tools have been developed to investigate axonal transport across a multitude of models [7]. For instance, the regulation of fast axonal transport has been studied in cultured primary neurons isolated from rodents [8], horses [9] and *Drosophila melanogaster* [10], in human-derived neurons [11], as well as in *ex vivo* preparations [12], sections of the giant axon of squid [13] and through *in vitro* reconstitution assays [14]. Additionally, techniques to monitor slow axonal transport of cargoes, such as neurofilaments, are also available [15,16].

To study transport in a more physiological environment, several *in vivo* methods have also been established to enable tracking of individual axonal cargoes [17]; for example, trafficking can be imaged in *Drosophila* larvae and adults [18,19], different zebrafish [20,21] and *Caenorhabditis elegans* [22] neurons, as well as in rodent sciatic nerves [23], spinal cord and dorsal roots [24], retinal ganglion cells [25] and the brain [26]. Considering the selective vulnerability of neurons across different neurological diseases, designing complementary intravital imaging methods to allow comparative transport analyses across neuronal subtypes and anatomically distinct regions will be a useful addition to the axonal transport toolkit. For example, comparison between major nerve branches of the rodent forelimbs and hindlimbs would enable study of the role of trafficking in the diverse peripheral nerve pathologies identified in models of genetic neuropathy [27,28], Kennedy’s disease [29], spinal muscular atrophy [30,31] and ALS [32], as well as in the regeneration processes of distinct spinal cord segments (e.g., cervical versus thoracic) occurring post-injury [33]. Measuring axonal transport in different nerve branches could also provide a readout for systematic evaluation of treatments in these animal models and accelerate recovery studies specifically targeting forelimbs and hindlimbs.

We have previously developed and optimised a technique to capture individual cargo movement in the hindlimb sciatic nerves of live, anaesthetised mice [34], and used this approach to identify early and pharmacologically reversible deficits in transport in mouse models of ALS [35–38]. Here, we have adapted this procedure to monitor *in vivo* axonal transport in murine median and ulnar nerves. Through injection of a fluorescently labelled, atoxic, binding fragment of the tetanus neurotoxin into the forepaw, which is taken up at nerve terminals and sorted into neurotrophin-containing signalling endosomes [39,40], we show that individual cargoes can be imaged and tracked in axons of the rodent forelimb. With this method, we aim to facilitate comparative transport analyses in rodent disease models; we hope that this will improve understanding of pathomechanisms in diseases affecting peripheral nerves and facilitate testing of novel therapies targeting these pathologies.

## Materials and methods

### General

All animal work was carried out under license from the U.K. Home Office in accordance with the Animals (Scientific Procedures) Act 1986 (Amended Regulations 2012) and approved by the Queen Square Institute of Neurology Ethics Committee. All animal work was conducted at the Queen Square Institute of Neurology, London, U.K. Animals used in this study were euthanised by an overdose of anaesthetic or dislocation of the neck with sedation. The images and data in the present study were produced using three month-old male and female wild-type mice on a C57BL/6J genetic background. The median and ulnar nerves are easier to manipulate in larger mice, thus we recommend that the earliest timepoint for imaging is ≈ 1 month of age.

### Reagents, equipment and set-up

The tools and equipment required for injections, dissections, anaesthesia and live imaging have been reported in detail elsewhere [34]. Injections into the forepaw and surgical exposure of the median/ulnar nerves require a dissection scope (e.g., SMZ-171 Stereo Zoom microscope, Motic). Representative dissection images and video were taken using a DSK 500 dual head stereo microscope (Motic, PM5539B901) with attached Moticam 1080 HDMI digital camera (Motic, MC1080). The binding fragment of tetanus neurotoxin (H_C_T) was expressed in *Escherichia coli*, purified and labelled with AlexaFluor 555 C2 maleimide (Life Technologies, A20346) as previously described [41].

The following primary antibodies were used: anti-choline acetyltransferase (ChAT, rabbit, Abcam, ab178850/RRID: AB_2721842, 1:250), anti-neurofilament (2H3, mouse, Developmental Studies Hybridoma Bank [DSHB], RRID: AB_531793, 1:250), anti-pan-synaptic vesicle 2 (SV2, mouse, DSHB, RRID: AB_2315387, 1:50) and anti-*β*III-tubulin (Tuj1, chicken, Abcam, ab41489/RRID: AB_727049, 1:250).

The following fluorescent antibodies and toxin were used: AlexaFluor 488 anti-rabbit IgG (donkey, Life Technologies, A21206, 1:500), AlexaFluor 647 anti-chicken IgG (Goat, Life Technologies, A21449, 1:500), AlexaFluor 647 anti-mouse IgG (donkey, Life Technologies, A31571, 1:500) and AlexaFluor 488 α-bungarotoxin (α-BTX, Life Technologies, B13422/RRID: AB_2617152, 1:1000).

The following items were used for live imaging: Zeiss LSM 780 NLO confocal microscope, oil immersion 63× objective (Zeiss Plan Apo 63×/1.4 Oil DIC II), anti-vibration table (Smart Table ST-UT2 Series, Newport), environmental chamber (Zeiss XL multi S1 DARK LS, 411857-9420-000), computer with microscope control and image acquisition software (Zen System 2012, 410135-1003-120), microscope stage for imaging of small rodents (custom made for Zeiss LSM 780, Digital Pixel), glass coverslips (22 mm × 64 mm, thickness 1.0, VWR International, 631-0142) and anaesthetic chamber with mask.

The following confocal settings were used for *in vivo* imaging: laser wavelength 561 nm, laser power 1%, emission range 570–624 nm, pinhole size 1-3 AU, gain (master) 600–900, frame size 1024 × 1024, scan speed 10 (pixel dwell 0.64 µsec), scan average 8, line mode, single-plane scanning. The following confocal settings were used for immunohistology: laser wavelengths: α-BTX and ChAT 488 nm, H_C_T 561 nm, SV2/2H3 and Tuj1 633 nm, laser power 0.2–1.0%, pinhole size 1 AU, gain (master) 600–750, frame size 1024 × 1024, scan speed 10 (pixel dwell 0.64 µs), scan average 4, line mode, z-slice interval 2.5–5 µm.

### Tissue dissection and staining

Wholemount forelimb lumbrical muscles and sciatic nerves were dissected, processed and stained as previously described [42–44].

### Axonal transport analysis

Axonal transport of signalling endosomes was imaged in the median/ulnar nerves and sciatic nerve as described previously [34]. To increase the likelihood of imaging motor neurons, axons with larger calibres were prioritised for imaging [37]. Confocal .czi files were uploaded to ImageJ (http://rsb.info.nih.gov/ij/) and the dynamics of individual endosomes manually tracked using the TrackMate plugin [45] and previously reported criteria [37]. For comparison of axonal transport in median/ulnar nerves with sciatic nerves, different mice were used (i.e*.*, both nerve preparations were not imaged in the same animal).

### Statistical analysis

Data were assumed to be normally distributed unless evidence to the contrary were provided by the Kolmogorov–Smirnov test for normality, while equal variance between groups was assumed. Normally-distributed data were statistically analysed using unpaired *t*-tests. All tests were two-sided and an α-level of *P* < 0.05 was used to determine significance. GraphPad Prism 9 software (version 9.5.0) was used for statistical analyses and data visualisation.

## Injection, dissection and transport protocol

### Forepaw injection with fluorescent H_C_T

**Figure 1:**
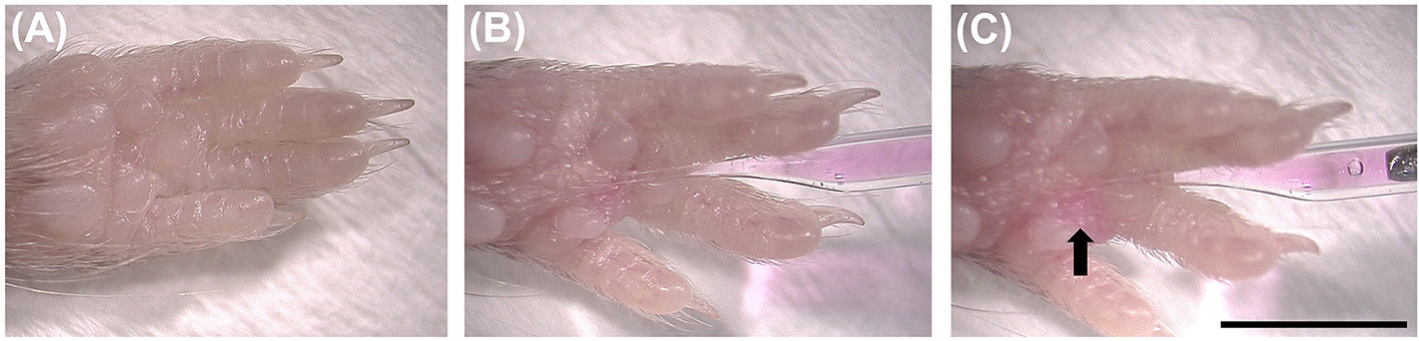
Injection of fluorescent atoxic tetanus neurotoxin binding fragment (H_C_T) into the forepaw (**A**) Anaesthetise the mouse and position the forepaw for injection, ventral-side upwards. (**B**) Use forceps (not shown) to gently hold and lift a small piece of skin in the middle of the paw. With a pulled, glass micropipette at a shallow, almost horizontal angle (i.e*.*, 10–20°) inject ≈ 1–2 μl H_C_T-555 (2.5–5 μg) into the paw through the lifted skin. (**C**) After successful injection, a small, coloured bulge containing H_C_T-555 can be seen (arrow). A left forepaw is being injected in these images. Scale bar ≈ 5 mm and is representative for all panels.

Using isoflurane gas and a heating pad to maintain body temperature, anaesthetise the mouse and position the animal on its back, ventral-side upwards. Confirm loss of sensation by checking the paw withdrawal reflex, and pay close attention to the anaesthesia level during the procedure. Extend the forelimb to the side of the body and use medical tape across the medial aspect of the limb to help position the forepaw (Figure 1). Gently hold and lift a small piece of skin in the middle of the paw using forceps. With a pulled, glass micropipette inject 2.5–5 μg H_C_T-555 in a volume of 1–2 μl into the paw through the lifted skin, avoiding the footpads. Performing injections under a dissection microscope is recommended to ensure accuracy of injection site and aid reproducibility. To avoid damaging the underlying musculature, inject at a shallow angle from the plane of the table (i.e*.*, 10–20°). A small bulging region containing H_C_T-555 can be seen in the paw immediately after a successful injection. Care must be taken to avoid multiple injections into this sensitive area. The use of fine micropipettes ensures that the procedure is minimally invasive and causes no overt pain responses in the animal after regaining consciousness. After injection move the mouse to an isolated cage and monitor recovery for up to 30 min; animals usually fully recover within a few minutes.

### Exposure of the median and ulnar nerves

**Figure 2:**
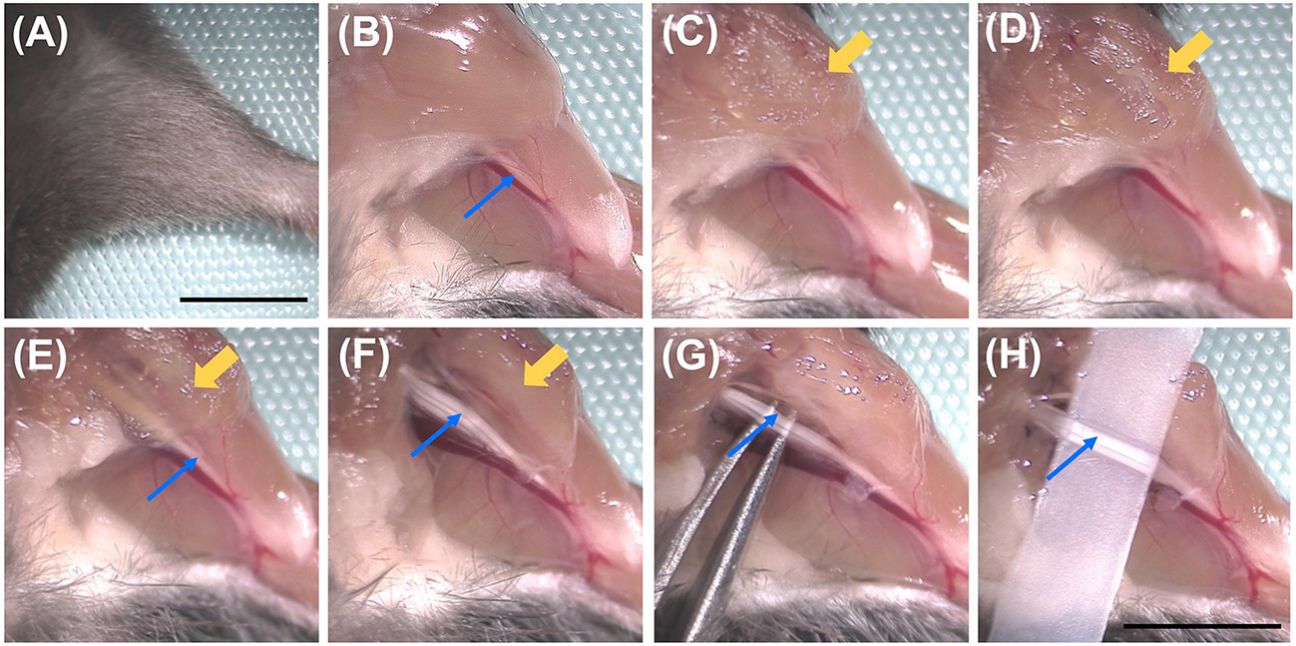
Exposure and preparation of the median and ulnar nerves for imaging (**A**) Position the anaesthetised mouse to expose the ventral side of the forelimb. The forelimb is extended to the side of the body at an angle of ≈ 90° from the midline. (**B**) Remove the skin to expose the median and ulnar nerves (blue arrows), which are adjacent to and parallel with the brachial artery. (**C–F**) Use forceps to lift the pectoral muscles (yellow arrows) that obscure the proximal portion of the median and ulnar nerves. With a small surgical scissor, cut away the muscles at a shallow, almost horizontal angle (to the table). Several muscle layers can be cut away step-by-step to expose a larger region of the nerves for imaging. (**G**) Add pre-warmed saline to restrict desiccation of the nerves. With curved forceps, gently separate the median and ulnar nerves from underlying connective tissue. (**H**) Carefully thread a small, flat piece of plastic/tape under the nerves to aid imaging. Left median/ulnar nerves are being exposed in these images. Scale bars ≈ 5 mm. The scale in panel (A) is representative of panels (B–E), and the scale in panel (H) is representative of panels (F) and (G).

Four- eight-hour post-injection and under terminal anaesthesia, using a dissection microscope expose and prepare the median and ulnar nerves for *in vivo* imaging (Figure 2 and Supplementary Video S1). This should be performed on a heating mat to minimise heat loss during surgery, not only for animal welfare, but because temperature impacts the rate of transport [46]. Position the anaesthetised mouse to expose the ventral side of the forelimb. Using tape across the paw, extend and fix the forelimb to the side of the body at an angle of ≈ 90° from the midline and remove the overlying skin (Figure 2A,B). The median and ulnar nerves can be seen next to the brachial artery (Figure 2B). Use forceps to gently lift up the pectoral muscles that obscure the proximal parts of the median and ulnar nerves. With a small surgical scissor at a shallow, almost horizontal angle, cut and remove bits of muscle (Figure 2C–E). Several layers of muscle can be sequentially cut away to expose a larger region of the median and ulnar nerves for imaging (Figure 2F). Pay extra attention to avoid damaging large blood vessels, as this will result in rapid blood pooling in the imaging area and the need to euthanise the mouse. Once the muscles are cleared, apply pre-warmed saline to the nerve to avoid desiccation. Using curved forceps, gently separate the median and ulnar nerves from the underlying connective tissue (Figure 2G). Carefully thread a small, flat piece of plastic (e.g., folded magic tape) under the nerves to aid imaging by providing clear separation between the nerve and underlying tissue (Figure 2H). The plastic should be sufficiently wide and strong to hold the nerves against the coverslip and thus maintain focus when imaging.

### Imaging axonal transport of signalling endosomes in median and ulnar nerves

**Figure 3:**
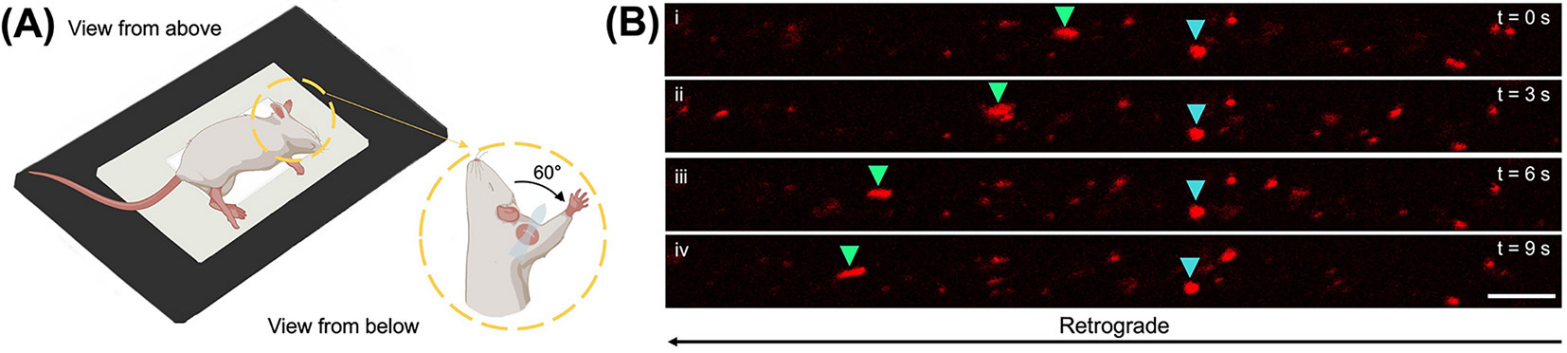
*In vivo* imaging of median and ulnar nerves to analyse retrograde axonal transport of signalling endosomes (**A**) Position the median and ulnar nerves on the stage of an inverted, confocal microscope and image at regular intervals (e.g., every 0.1–3.5 s). To ensure effective contact of the median/ulnar nerves with the coverslip, the forelimb with exposed nerves is extended towards the head and fixed in place using tape at an angle of ≈ 60° from the midline. The schematic was created with BioRender (https://biorender.com). (**B**) Individual, H_C_T-loaded signalling endosomes (red) being retrogradely transported can be tracked along axons. Panels i to iv show a series of single-plane confocal images taken every 3.0 s (progressing from top to bottom). Individual endosomes (e.g., coloured triangles) can be tracked across multiple, consecutive images to assess retrograde axonal transport dynamics. N.B*.*, the cyan arrow highlights a paused endosome. Scale bar = 5 μm and is representative for all immunofluorescent images. See also Supplementary Figure S1.

Under continued terminal anaesthesia, move the mouse to the pre-warmed environmental chamber of the inverted confocal microscope and position the mouse on its side on the microscope stage (Figure 3A). To provide efficient contact of the median and ulnar nerves with the coverslip, the exposed forelimb is extended towards the head and fixed in place using tape at an angle of ≈ 60° from the midline (Figure 3A inset and Supplementary Figure S1). Adjust the mouse body position to minimise the effect of breathing movements, which can hinder transport analyses. H_C_T is taken up into a dense population of axons, which can be identified by their linear arrangement of fluorescence and careful adjustment of the focal plane.

Image at regular intervals to visualise individual, H_C_T-positive signalling endosomes being retrogradely transported along axons (Figure 3B) [34]. The rate at which images are taken can vary from 0.1 to 3.5 s, depending on experimental requirements, such as the cargo being imaged (e.g., slower moving mitochondria or faster signalling endosomes) and tracking method (e.g., manual or semi-automatic). For signalling endosomes, we predominantly use a frame rate of 3 s for 100–200 frames, which allows a field of view (≈ 67 µm × 26 µm) that can accommodate multiple, clearly distinct axons. Axons can be in close proximity or overlapping, thus we assess transport in regions within a single field of view that are visibly separated or in different regions of the nerve; this ensures that we track endosomes in at least three separate axons. Fluorescent reporter strains, such as ChAT.eGFP mice, can be used to visualise individual motor axons [37]. The 3 s frame rate also permits line averaging of 8 to produce images with high quality to confidently identify individual endosomes (based on size, signal intensity and trajectory) as they travel from one end of the axon to the other. A faster frame rate will make this easier, as there is less distance travelled between frames, but there will be a small trade-off in image quality.

Once taken, image time series can be manually or semi-automatically tracked and analysed using the TrackMate plugin in ImageJ to reveal trafficking dynamics [45]. Slower frame rates (such as 3 s) require manual tracking of cargoes, as the TrackMate software struggles to follow the same endosome in densely populated axons, which can be exacerbated by breathing artefacts and slow shift of the nerve across the coverslip. However, semi-automatic tracking is possible with image series taken using a faster frame rate (e.g., 0.3 s), ensuring accurate linking of endosomes between frames [47]. Importantly, the frame rate does not influence the observed average endosomes speeds [37,47]. As an alternative to tracking, kymographs can be generated and used for transport analysis [48].

## Results and discussion

### H_C_T efficiently localises to the neuromuscular system

We have previously shown that H_C_T injected into tibialis anterior muscle localises to neuromuscular junctions (NMJs) [34,35]. This occurs within a few hours and begins to slowly decline 4–8 h-post injection due to H_C_T uptake into axons of the sciatic nerve. Upon inspection of sciatic nerve cross-sections, it was determined that ≈ 80% of H_C_T-positive axons expressed ChAT, indicating that most neurons containing fluorescent endosomes are motor rather than sensory in function [35]. We have also observed rapid H_C_T accumulation and uptake at NMJs *in vivo* in the epitrochleoanconeus muscle of the forelimb [49].

**Figure 4:**
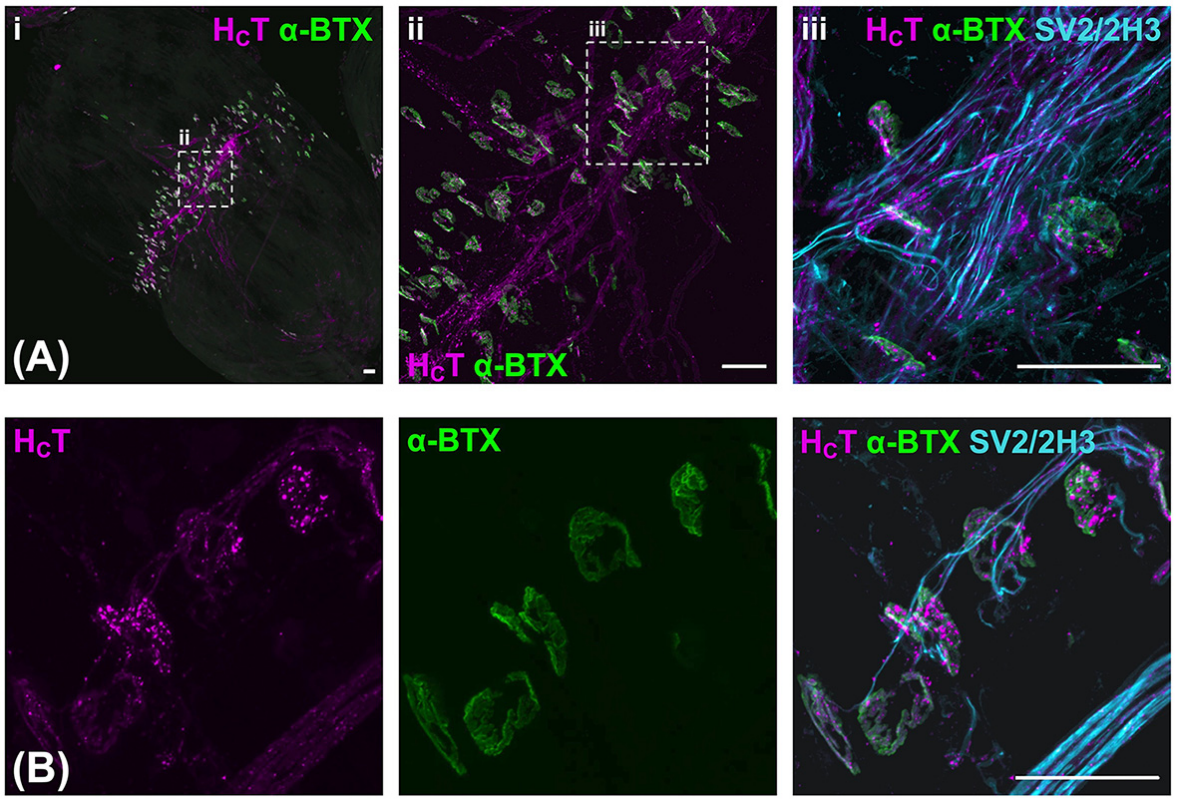
H_C_T is efficiently taken up at motor nerve terminals after injection into the forepaw Forelimb lumbrical muscles were dissected 4 h post-H_C_T injection and fixed in 4% PFA for 10 min before wholemount staining with α-BTX (green) and anti-SV2/2H3 antibodies (cyan). (**A**) Images of a forelimb lumbrical muscle showing typical H_C_T (magenta) uptake at neuromuscular junctions (α-BTX, green) and localisation in motor axons (SV2/2H3, cyan). i depicts a 20× tile scan image, ii depicts a 20× single image, and iii depicts a 63× single image. The dashed boxes in panels i and ii represent the imaging regions of panels ii and iii, respectively. (**B**) 63× scanning images showing that H_C_T is found at motor nerve terminals and axons. All images are collapsed, maximum-intensity z-stack projections. Scale bar = 50 μm.

To determine whether H_C_T is similarly taken up at NMJs in muscles of the injected forepaw, forelimb lumbricals were dissected 4 h post-H_C_T injection, followed by fixation and staining. The major benefit of the lumbrical muscles is that they can be wholemount dissected and processed, such that uptake of the fluorescent probe can be visualised across the entire muscle. Performing immunofluorescent staining with α-bungarotoxin (α-BTX) to label post-synaptic acetylcholine receptors and anti-SV2/2H3 antibodies to identify neurons, we observed extensive co-localisation of H_C_T with NMJs and their innervating motor axons (Figure 4). In fact, there appeared to be complete and specific coverage of the neuromuscular system with this fluorescent probe, suggestive of extremely efficient uptake of H_C_T into motor neurons innervating lumbrical muscles of the forepaws.

### H_C_T is transported into axons of median and ulnar nerves

**Figure 5:**
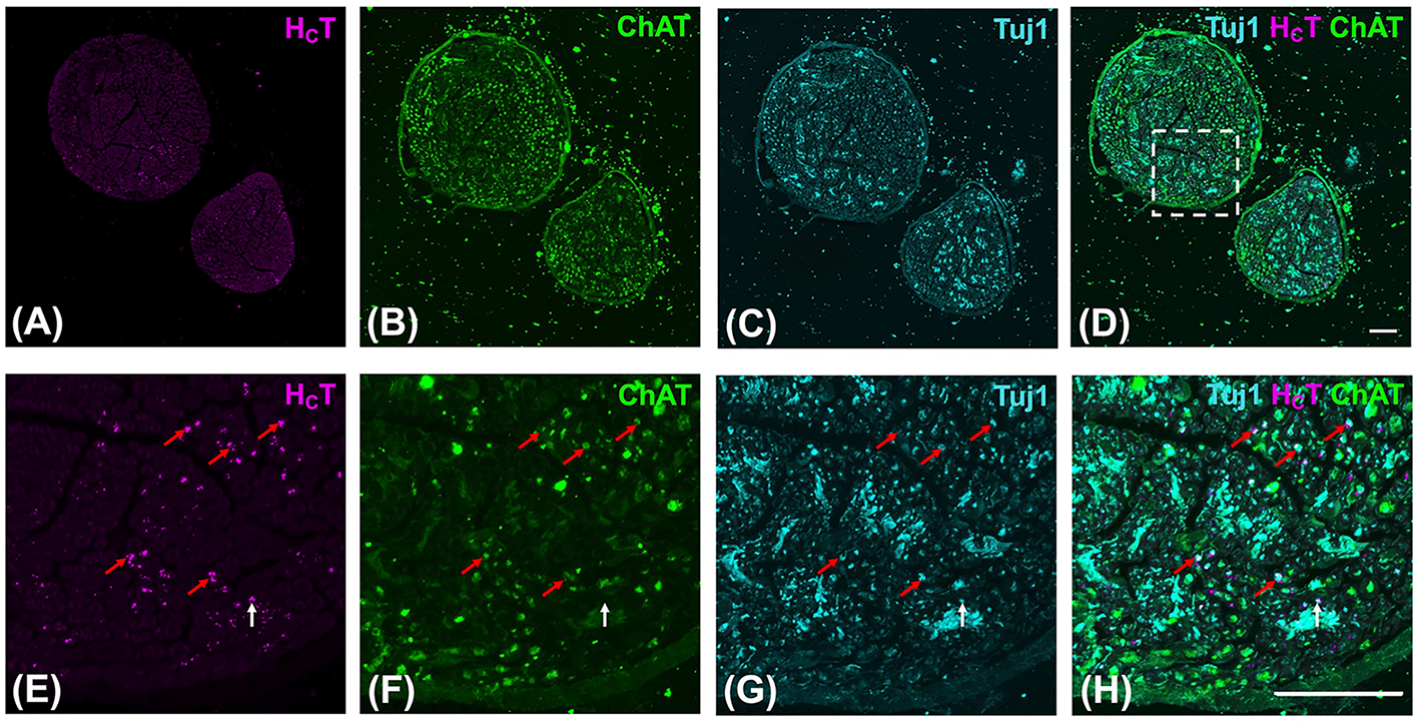
H_C_T is transported into median and ulnar axons after injection into the forepaw (**A–D**) 20× tile scanning images of 10 µm-sectioned median/ulnar nerves giving a typical view of H_C_T (magenta) localisation in axons. Median/ulnar nerves were dissected 4 h post-H_C_T injection into the forepaw. The nerves were then fixed in 4% PFA for 48 h and transverse sections cut using a cryostat before staining with anti-Tuj1 (cyan) to label all axons (**C,G**) and anti-ChAT (green) to stain only motor axons (**B,F**). The dashed box in panel (D) represents the imaging regions of panels (E–H). The median nerve is the larger of the two nerves. (**E–H**) 63× scanning images showing that H_C_T is transported into axons. H_C_T predominantly localises to motor axons (red arrows: Tuj1-positive, ChAT-positive) and is rarely seen in sensory axons (white arrows: Tuj1-positive, ChAT-negative). All images are collapsed, maximum-intensity z-stack projections. Scale bars = 50 μm.

To confirm that the interaction of H_C_T with the neuromuscular architecture in forelimb lumbrical muscles results in transport into motor axons, we performed immunofluorescence analysis of median and ulnar nerves dissected 4 h after H_C_T injection into the forepaw (Figure 5). Fixed and transverse-sectioned nerves were stained with anti-Tuj1 to label all axons (Figure 5C,G) and anti-ChAT to highlight motor-specific axons (Figure 5B,F). H_C_T predominantly localised to motor axons (red arrows: Tuj1-positive, ChAT-positive) and was only rarely seen in sensory axons (white arrows: Tuj1-positive, ChAT-negative) (Figure 5H). This is consistent with the previous assessment of the sciatic nerve [35] and suggests that H_C_T is efficiently taken up into motor axons of the median and ulnar nerves upon injection into the forepaw. The forepaw lumbrical muscles that insert on the second and third digits are innervated by the median nerve, while those inserting on the fourth and fifth digits are supplied by the ulnar nerve [50]. For reference, the first digit is the shortest in length and equivalent to the human thumb in position. We observed uptake of the probe in all lumbricals of the forepaw, explaining why H_C_T could be observed in both median and ulnar nerves. Nonetheless, it is likely that the probe also targeted other muscles after injection (e.g., interossei muscles).

### Axonal transport dynamics of signalling endosomes are similar between major forepaw and hindpaw nerves

**Figure 6:**
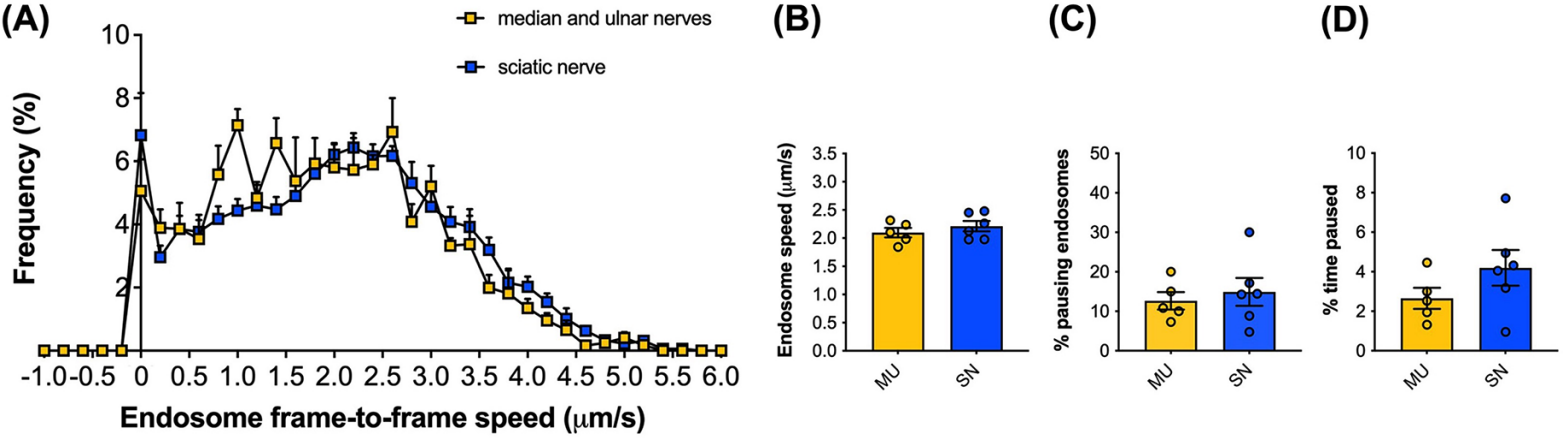
Signalling endosome transport dynamics are similar between median/ulnar (forelimb) and sciatic nerves (hindlimb) in 3-month-old WT mice (**A**) Frame-to-frame speed distribution curves indicate that baseline retrograde trafficking dynamics of signalling endosomes are similar between the median/ulnar nerves (yellow) and the sciatic nerve (blue) in three month-old wild-type mice. (**B–D**) Mean endosome speeds (B), % pausing endosomes (C) and % time paused (D) are calculated per animal and show no significant differences (*P* > 0.05, unpaired *t*-tests) between the median/ulnar nerves (MU, yellow) and the sciatic nerve (SN, blue). The sciatic nerve data and median/ulnar nerve data were generated from different mice. Means ± SEM are plotted for all figures; *n* = 5–6.

To determine whether the baseline transport dynamics of signalling endosomes are similar between sciatic nerves and median/ulnar nerves, we performed imaging and analyses with H_C_T in 3-month-old wild-type mice. Frame-to-frame speed distribution curves indicate that baseline retrograde trafficking of signalling endosomes is similar between nerves (Figure 6A). This finding was supported by the comparison between the average endosome speed, the percentage of pausing endosomes, and the percentage of time that endosomes paused per animal (Figure 6B–D). No significant differences in transport were observed between the median/ulnar nerves and the sciatic nerve. This is consistent with earlier work indicating that signalling endosome trafficking speeds are comparable between sciatic nerve motor axons innervating different muscles of the hindlimb (i.e*.*, gastrocnemius, soleus and tibialis anterior) [47]. The transport speeds we observed in the median/ulnar nerves also support our previous finding that axonal transport of H_C_T-positive signalling endosomes is faster *in vivo* than *in vitro* [41]. Lack of myelination and the correct balance of target-derived signals, such as neurotrophic factors, in the artificial *in vitro* environment are possible causes for the difference [17].

By analysing and comparing transport in GFP-positive motor axons and GFP-negative axons in the sciatic nerve of live ChAT-eGFP mice, we have previously determined that signalling endosomes are transported more quickly in motor than sensory axons [37], which is also observed with mitochondria and lysosomes in human induced pluripotent stem cell-derived neurons [51]. Furthermore, the H_C_T-positive motor axons could also be identified by their calibres, which are 2- to 3-fold larger than their sensory counterparts. Given the efficient targeting of the neuromuscular system by H_C_T in forelimb lumbricals (Figures 4 and 5), our preferential imaging of larger calibre nerves, and the similarity in endosome dynamics between median/ulnar and sciatic nerves (Figure 6), it is highly likely that our transport analysis has been predominantly, if not solely, performed in motor rather than sensory axons of the median/ulnar nerves.

Compared with our previously described method for imaging signalling endosomes in sciatic nerves [34], this adapted approach in the median and ulnar nerves has the main limitation that it is more technically challenging; the surgical area in the forelimb is smaller than the sciatic nerve region and the median/ulnar nerves are situated adjacent to the brachial artery, which, if accidentally damaged, will result in having to euthanise the mouse. The procedure thus requires a dissection microscope, unlike sciatic nerve exposure, and additional time (≈10 min). We thus recommend practicing on spare euthanised mice until the procedure can be efficiently performed without error. Moreover, given their position closer to the body, it can be challenging to locate the median and ulnar nerves in the exposed forelimb once it has been secured to the microscope stage (Figure 3A inset and Supplementary Figure S1). To maintain contact between the nerves and the coverslip, the arm must be securely fastened to the stage using strong tape. Again, this should be practiced with euthanised mice; by removing the microscope stage, different body, arm and tape positions can be tested for the best nerve-coverslip contact. Despite these difficulties, the adapted technique possesses the advantage that H_C_T uptake can be more easily analysed in wholemount lumbrical muscles of the forepaw. This is not readily achievable using the sciatic nerve approach in which the probe is injected into large hindlimb muscles (e.g., tibialis anterior).

## Conclusions

We describe a method for *in vivo* imaging of axonal transport in nerves of the rodent forelimb. By injecting a fluorescently labelled and non-toxic fragment of tetanus neurotoxin into the forepaws, signalling endosomes can be identified in intact peripheral axons of median and ulnar nerves. Upon injection, H_C_T is efficiently taken up at NMJs and into motor nerve terminals, before being transported predominantly into motor axons in median and ulnar nerves. Applying this technique, baseline trafficking dynamics of signalling endosomes were found to be similar between the median/ulnar nerves and the sciatic nerve in adult wild-type mice. This approach can be adapted to analyse additional cargoes, such as mitochondria, either individually or simultaneously [23], which will be advantageous for studying the important interactions between axonal organelles [52]. Together with comparative pathological assessments across limbs in rodent models of disease, for example, NMJ innervation and functional measurements [27,53,54], we believe that axonal transport analyses in both sciatic and median/ulnar nerves will provide a powerful approach to better understand the temporal and spatial progression of pathology in diseases and traumas affecting peripheral nerves. This will be particularly useful in mouse models in which there are reported distinctions in sensory and motor nerve vulnerability between limbs, such as genetic neuropathy [27,28], and also for determining whether similar patterns of peripheral nerve pathology observed in human diseases, such as Guillain-Barré syndrome [55], are replicated in rodent models.

## Supplementary Material

Supplementary Figure S1

Supplementary Video S1

## Data Availability

All data are provided in the main text.

## Open Access

Open access for this article was enabled by the participation of University College London in an all-inclusive *Read & Publish* agreement with Portland Press and the Biochemical Society under a transformative agreement with JISC.

## Abbreviations

α-BTX: α-bungarotoxin
ALS: amyotrophic lateral sclerosis
ChAT: choline acetyltransferase
H_C_T: binding fragment of tetanus neurotoxin
NMJ: neuromuscular junction
SV2: synaptic vesicle 2
Tuj1: *β*III-tubulin
2H3: neurofilament
