# Supplementary material for: In vivo imaging of axonal transport in peripheral nerves of rodent forelimbs
Source: Neuronal Signal. 2023 Jan 19;7(1):NS20220098. doi: 10.1042/NS20220098 (PMC9867938; doi:10.1042/NS20220098)
Supplement: Supplementary Figure S1 [file NS-2022-0098_supp.pdf]

In vivo imaging of axonal transport in peripheral nerves of rodent forelimbs

Qiuhan Lang[1], Giampietro Schiavo[1,2] and James N. Sleigh[1,2*]

Affiliations:

[1] Department of Neuromuscular Diseases and UCL Queen Square Motor Neuron Disease Centre, Queen Square Institute of Neurology, University College London, London WC1N 3BG, UK.

[2] UK Dementia Research Institute, University College London, London WC1E 6BT, UK.

[*] Corresponding author:

j.sleigh@ucl.ac.uk

Tel: +44(0)20 3108 2722

ORCID

Lang: 0000-0003-3880-1105

Schiavo: 0000-0002-4319-8745

Sleigh: 0000-0002-3782-9045

Supplementary Information

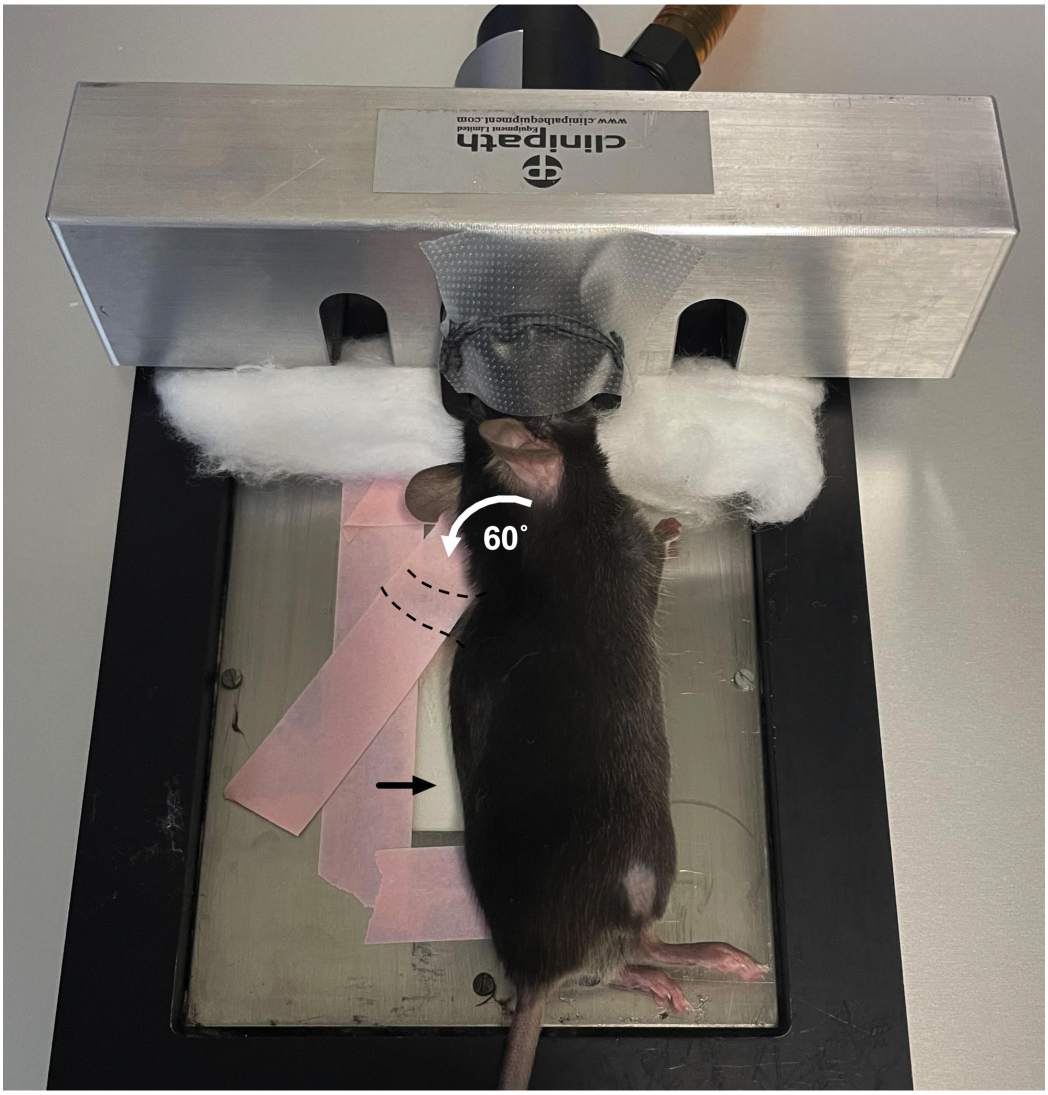

Supplementary Figure 1. Securing the forelimb for imaging. To ensure effective contact of the median/ulnar nerves with the coverslip (arrow), the forelimb to be imaged (dashed lines) is extended towards the head and fixed in place at an angle of ≈ 60˚ from the midline. Several bits of strong tape may be required. *N.B.*, the stage has been removed from the microscope to facilitate taking of the image. See also **Figure 3**.
