# Supplementary material for: In vivo imaging of axonal transport in peripheral nerves of rodent forelimbs
Source: Neuronal Signal. 2023 Jan 19;7(1):NS20220098. doi: 10.1042/NS20220098 (PMC9867938; doi:10.1042/NS20220098)
Supplement: Supplementary Video S1 [file NS-2022-0098_supp1.zip › NS-2022-0098_suppsm.pdf]

Supplementary Video 1. Preparation of the median and ulnar nerves for imaging. This video shows the preparation of mouse median and ulnar nerves for imaging. First, remove layers of the pectoral muscles overlaying the proximal portion of the nerves (00:00 to 02:17). Apply pre-warmed saline (02:22), before carefully separating the nerves from underlying connective tissue using curved forceps (03:00). Thread a narrow and thin piece of plastic (e.g., folded magic tape) beneath the nerves to aid imaging (03:20). A three month-old male is presented in the video. See also **Figure 2**, which can be used to discern scale. Video run time is 03:45 and there is no audio.
